# 

**Published:** 1851-07

**Authors:** 


Dr. Reid's communication will appear in our next No. Several new works have been received, which will be noticed as soon as practicable.

